# Synchronization of senescence and desynchronization of flowering in *Arabidopsis thaliana*

**DOI:** 10.1093/aobpla/plaa018

**Published:** 2020-05-09

**Authors:** Matin Miryeganeh

**Affiliations:** Center for Ecological Research, Kyoto University, Hirano, Otsu, Japan

**Keywords:** *Arabidopsis*, QTL, reproductive synchrony, RILs, senescence, sequential seeding

## Abstract

In a recent publication, we proposed that adjusting lifespan in order to synchronize senescence is important for timing of reproduction, and we quantified the synchrony of reproductive timing relative to germination timing. Here, in a second sequential seeding experiment (SSE), the germination timing of *Arabidopsis thaliana* accessions was manipulated and plants were then grown under two different temperature regimes. Life stage traits of plants in each temperature regime were analysed and it was evaluated whether the cohorts were grouped according to age and/or environmental conditions. While flowering-related traits showed desynchrony among cohorts, striking synchrony in the timing of senescence among cohorts for each group was found. A quantitative trait locus (QTL) analysis using a genotyped population of ‘Cvi/Ler’ recombinant inbred lines (RILs) was then conducted. Novel and known loci were assigned to flowering and senescence timing. However, senescence synchrony resulted in low variation in senescence time and weak QTL detection for flowering termination. Overlapping flowering and senescence genes with loci affecting either of those traits were found and suggest a potential interdependency of reproductive traits.

## Teaser

In this study we looked at whether initiation of flowering and flowering termination are individually synchronized or not, over the course of a growing season for four early-flowering arabidopsis accessions. We found that groups of same age plants sown 4 weeks apart are not synchronized for initiation of flowering time, but are synchronized when it comes to senescence. A genetic control of senescence (or termination of flowering) may have implications for crop improvement, as one might be able to change the balance between the production of flowers and seeds and resource allocation to leaves during the process of seed loading.

## Introduction

Plants synchronize reproduction in response to environmental factors so as to increase survival of offspring (Ims 1990). Offspring are produced under suitable environmental conditions, such as temperature, moisture and light levels (Rathcke and Lacey 1985; Morgan *et al.* 2011), seasonal indicators that signal adequate resource availability. Central tendency in flowering date among individuals determines the degree of flowering synchrony of a plant species (Bawa 1983). Synchronization of flowering is commonly observed in plants (Schmitt *et al.* 1987; Salome *et al.* 2011). This is because plants are required to synchronize not only with other conspecifics, e.g. in the case of outcrossing species to exchange pollen, but also with a specific season for successful seed maturation and optimal seed dispersion (Stinchcombe 2004). Annual plants complete their life cycle in a single year and their fitness is determined by a single reproductive event (King and Roughgarden 1982; Debieu *et al.* 2013). Therefore, synchronization of life-history decisions with timing and length of the optimal season is expected to be particularly strong in annuals, mainly because it is the major determinant of final seed yield (Montesinos-Navarro *et al.* 2012; Debieu *et al.* 2013). Many studies have shown that the degree of flowering synchrony is an ecological trait that affects a plant’s reproductive success (Rathcke and Lacey 1985; Zimmerman 1990). However, floral senescence synchrony has not received much attention and the effects of environmental changes on patterns of senescence synchrony and subsequent seed production are not well-understood.

In our previous study (Miryeganeh *et al.* 2018), we developed an experimental framework under natural conditions to quantify flowering and senescence synchronization using four accessions of arabidopsis, two early flowering (C24, Ler-1) and two late flowering (Lov-5, Tamm-2). Seven cohorts of each ecotype were germinated at 1-week intervals starting in October, so that later cohorts experienced lower temperatures and a reduced photoperiod (i.e. reduced photothermal units (PTUs)). Photothermal unit is a measure of temperature and photoperiod for growth perceived by plants (Wilczek *et al.* 2009). All four ecotypes used in that study showed senescence synchrony, regardless of when they were planted. On the other hand, flowering initiation and bolting were desynchronized among cohorts of only early-flowering accessions and were synchronized among cohorts of only late-flowering accessions. Considering that late-flowering accessions are reported to require strong vernalization before flowering, in contrast to early-flowering accessions, which flower relatively early even without vernalization (Lee and Amasino 1995; Sanda and Amasino 1995; Salome *et al.* 2011); the results of that study therefore emphasized the importance of vernalization in vernalization-dependent (i.e. late flowering) ecotypes, but not in less vernalization-dependent (i.e. early flowering) ecotypes.

Most early-flowering accessions have low FLC expression and/or a non-functional FRI gene (Shindo *et al.* 2005), which act synergistically to repress flowering. Vernalization releases the repression of flowering by FLC by decreasing gene expression through histone remodelling of the FLC locus (Sung and Amasino 2005). In our previous study, desynchronization of flowering in early-flowering accessions (C24 and Ler-1) led us to conclude that when the FRI–FLC system has a weak influence on flowering time (meaning in early-flowering accessions), the timing of flowering is better explained by PTU-dependent regulation. Calculating the vegetative PTU (PTU from the time of germination to flowering initiation) also showed constant vegetative PTU values across the seven cohorts of early-flowering accessions, regardless of the length of the period. The number of rosette leaves at bolting also was found to be more or less constant among cohorts in the early-flowering accessions, which implied that older cohorts of these accessions flowered even before winter—without vernalization—when they perceived a constant amount of PTU and produced a constant number of leaves. That study suggested that under uniform environmental conditions, arabidopsis plants synchronize senescence according to the seasonal environment, even if they differ in age. The early-flowering accessions appeared to accomplish this by changing the length of the flowering period.

**Figure 1.**
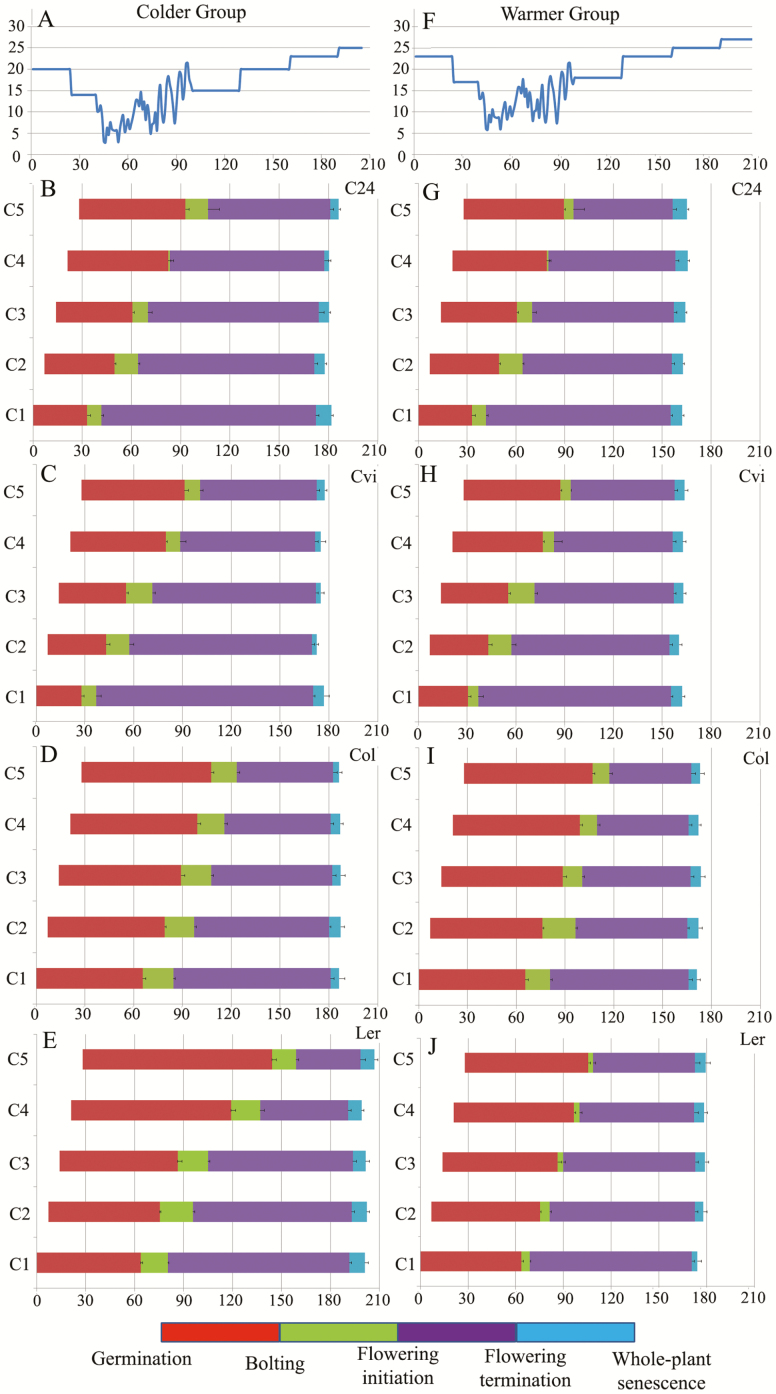
Results of SSE for four accessions of arabidopsis. Phenological responses of the five cohorts for (B) C24, (C) Cvi-0, (D) Col-0 and (E) Ler-1 in Colder Group and for (G) C24, (H) Cvi-0, (I) Col-0 and (J) Ler-1 in Warmer Group are indicated by bars representing the periods between germination and the timing of four successive reproductive periods. Colours correspond to those in the bars at the bottom of the figure. The *x*-axis shows the calendar date and the *y*-axis in the top panel shows the temperature for Colder Group (A) and Warmer Group (F).

In an effort to better understand the influences of environment and more specifically of PTU upon senescence synchrony, here a second sequential seeding experiment (SSE) was performed, by growing five cohorts of only early-flowering arabidopsis accessions, with members of each cohort having the same age. Cohorts (C1–C5 here after; Fig. 1) were seeded at 1-week intervals, meaning that each cohort was 7 days older than the preceding one; therefore, each cohort experienced fewer PTUs than those before. Eight replicates from each cohort were then transferred to each of two greenhouses with different temperature settings (referred as ‘Colder Group’ and ‘Warmer Group’), to compare reproductive synchrony of individuals within and between two groups. Four early-flowering arabidopsis accessions (C24, Cvi-0, Col-0 and Ler-1) were employed to track adjustment of flowering period and senescence synchrony.

Under the hypothesis that plants senesce in response to seasonal cues, plants of the same age that experience different seasonal environments should senesce at different times, while plants of different ages experiencing the same environment should senesce at the same time. Life event synchrony has to be evaluated in relation to germination timing. To do this, we quantified the degree of synchrony in reproductive events, by quantifying variation of the reproductive phenology between cohorts relative to that of germination timing. This was measured by calculating SIg, an index developed in our previous work that calculates synchronization index by considering germination timing (Miryeganeh *et al.* 2018). To obtain SIg (to evaluate phenological synchronization against variation in germination timing), one first calculates cohort means of an event, and then variances across cohort means.

$$\begin{array}{ll} SIg=\; & log_{2}[(variance\;of\;germination\;timing)\\ & /(variance\;in\;timing\;of\;the\;event)]. \end{array}$$

SIg > 0 indicates synchrony (e.g. SIg = 1 and 2 correspond to halved and quartered variance, respectively), whereas SIg < 0 denotes desynchrony (e.g. SIg = −1 and −2 correspond to doubled and quadrupled variance, respectively) (Miryeganeh *et al.* 2018). 

Next, in an attempt to decipher the genetic architecture of studied traits, an experimental recombinant inbred line (RIL) population was used to perform quantitative trait locus (QTL) analysis, a statistical method that allows identification of specific regions of chromosomes, using molecular markers that contribute to variation in specific phenotypic traits (Falconer and Mackay 1996; Kearsey 1998; Lynch and Walsh 1998). Here, an RIL mapping population derived from a cross between Cape Verde Islands (Cvi) and Landsberg erecta (Ler) strains was analysed. This population was chosen because QTL mapping requires large phenotypic differences between parental ecotypes in order to be able to identify the underlying genetic basis for phenotypes, and the parental lines for this RIL displayed contrasting flowering time, senescence time and flowering period phenotypes in the SSE (Cvi and Ler accessions were the earliest and latest flowering and senescing accessions, respectively. Also they showed the shortest and longest overall flowering period, respectively; Fig. 1). In addition, mapping QTL requires a segregating population for which a genetic map and accurate phenotyping of the trait have been established and availability of a permanent mapping population between Cvi and Ler offered these advantages (Alonso-Blanco and Koornneef 2000). A previous QTL analysis using the same RILs (Ler-0 and Cvi-0) also has shown heritability for the flowering period (Luquez *et al.* 2006). Therefore, after finding strong seasonal-dependent senescence synchrony among cohorts in our previous SSE (Miryeganeh *et al.* 2018), here we attempted to do a QTL analysis at the same experimental garden used for that SSE, in order to explore the genetic architecture of studied traits (flowering initiation and flowering termination). The goal of this QTL experiment was to identify genetic regions potentially associated with reproductive synchrony and also to identify and locate QTL responsible for the phenotypic variation observed in reproductive timing.

## Materials and Methods

### Plant material and SSE

For sequential seeding, four early-flowering accessions of arabidopsis; i.e. C24, Cvi-0, Col-0 and Ler-1 were chosen. Seeds for all accessions were obtained from ‘The *Arabidopsis* Biological Resource Center (ABRC) - The Ohio State University’ (http://www.arabidopsis.org/abrc/). Sequential seeding experiment was designed to quantify the synchrony of reproductive events such as bolting, flowering initiation, flowering termination and whole-plant senescence, by seeding plants at designated intervals (Miryeganeh *et al.* 2018). In this study, for each accession, five age cohorts of plants were prepared by sowing seeds five times at 1-week intervals. For each cohort (C1–C5), seeds of all four accessions were sown at the same time each week. Therefore, each cohort was exactly 7 days older than the preceding one. The seeding dates for C1–C5 were 2, 9, 16, 23 and 30 December 2014, respectively. At each seeding date, 30–40 surface-sterilized seeds of each accession were sown individually on the surface of culture media in petri dishes with at least 2 cm away from each other. Each dish contained 50 mL of Murashige and Skoog medium. After being kept at 4 °C in darkness for 3 days, petri dishes from each cohort were moved to two incubators (GROWTH CABINET- HNM - S11, Koito Industries, Ltd, Japan)—as ‘Colder Group’ and ‘Warmer Group’—where on average they received 300 µM m^−1^ s^−1^ photosynthetically active radiation (PAR), during a 12-h photoperiod, day/night temperature of 20/20 °C in ‘Colder Group’ and 23/23 °C in ‘Warmer Group’ **[see****Supporting Information—Dataset Table 2****]**. After 10 days in the incubator, eight seedlings from each cohort in each group (in total 40 plants for five cohorts in each group) were randomly chosen and transferred to 10-cm diameter clay pots. Soil consisted of a coarse-grained loamy soil in the lower half of the pot and a 1:1 mixture of fine-grained pumice and peat in the upper half. A slow-acting fertilizer (Mag Amp K) was added according to manufacturer’s instructions (50 g per 12 L mixed soil; ratio of N:P:K:Mg = 6:40:6:15). After transplantation, all potted plants were successively transferred to two (one for each group) air-conditioned greenhouses (KOITOTRON: S-180, KI Holdings Co., Ltd, Kanagawa, Japan), at the Center for Ecological Research, Kyoto University, where they were subjected to two different temperature settings, described in **Supporting Information—Dataset Table 2**. Positions of accessions and cohorts within each greenhouse were randomized and the pots were placed at about 15 cm distances from one another. The reason for using greenhouses instead of the experimental garden for the current SSE was to manually make two environmental condition—using temperature as the most dominant variable among seasons—and calculate the final PTU received by plants and therefore be able to re-evaluate the reported environmental-dependent ‘senescence synchrony’ in our previous study which was done in a natural environment.

Throughout the experiment, soil surface temperature was recorded every 30 min using a HOBO Water Temp Pro v2 temperature logger (Onset Computer Corporation, Bourne, MA, USA). In each house, the logger was set just beneath the surface of the soil in a pot without a plant. Photoperiod records were obtained from the website of the National Astronomical Observatory of Japan (NAOJ, http://eco.mtk.nao.ac.jp/koyomi/dni/dni26.html). Plants were watered at planting to reduce mortality after transplantation and were also watered at least four times a week during the experiment until the day that all vegetative and reproductive parts were completely withered. Phenological traits were monitored and recorded daily throughout the entire experiment. Initial elongation of main stems >3 mm was considered bolting. Flowering initiation was defined as the day of first anthesis, i.e. opening of the first flower. Flowering termination was defined as the day that the last flower withered. Whole-plant senescence was defined as the day that all vegetative and reproductive parts were completely withered. To represent plant size, the number of leaves and diameter of the basal rosette at the time of bolting were recorded. The total number of fruits produced by each plant was also recorded. A complete list of phenological measurements is shown in **Supporting Information—Dataset Table 1**.

### PTU during vegetative and flowering periods

Vegetative period (the period from germination to flowering initiation) and flowering periods (the period between flowering initiation and termination) were quantified according to photothermal time (PTU, degree days (Granier *et al.* 2002)) as well. Photothermal unit for vegetative and flowering periods was calculated as the cumulative temperature and cumulative light over the basal threshold temperature (µ _b_) during the daytime and the daily photoperiod as a proportion of 24 h (λ _*i*_):

$$\begin{array}{l} PTU=\underset{i=g\;or\;fi\;}{\overset{fi\;or\;ft}{\sum }}\;\lambda_{i}(\mu_{Li}-\;\mu_{b}) \end{array}$$

where ‘photothermal time’ is expressed in PTUs (°C, daylight), and *i* spans the germination date (*g*) to the time of flowering initiation (*fi*) for the vegetative period or from *fi* to flowering termination (*ft*). λ _*i*_ is the daily photoperiod as a proportion of 24 h, counting only days with a mean temperature (µ) during daylight hours (µ _L*i*_), where µ _L*i*_ was greater than µ _b_. In this study, we applied µ _b_ = 3 °C, which has been reported as the optimized base temperature in modelling the developmental rate of Col-0 (Granier *et al.* 2002).

### Statistical analyses

For each accession, differences between cohorts in vegetative and flowering periods and in vegetative and flowering PTU were tested using Duncan’s multiple comparison tests at *P* < 0.001, using ‘multcomp’ in R software version 3.3.2 (R Core Team 2016). Correlation coefficients (*r*) and the number of rosette leaves for all cohorts during the vegetative period and during the interval between the flowering and vegetative periods for each accession were also calculated.

### Phenotyping of RILs

The RIL experiment was conducted in a natural environment using an outside experimental garden at the Center for Ecological Research, Kyoto University (34°58′N, 135°57′E, 150 m elevation), which has a temperate climate with moderate seasonal temperatures and photoperiodic changes. F10 seeds of a set of 105 RILs, derived from crosses between the laboratory strain, Ler, from northern Europe (Re’dei 1992) and accession Cvi, from the Cape Verde Islands (Lobin 1983), were used in this study. These RILs have been previously characterized for amplified fragment polymorphism and CAPS (cleaved amplified polymorphic sequences) markers (Alonso-Blanco *et al.* 1998). Seeds of these RILs were obtained from ‘The *Arabidopsis* Biological Resource Center (ABRC) - The Ohio State University’ (http://www.arabidopsis.org/abrc/). For each line, 80–100 surface-sterilized seeds were sown individually on culture medium in petri dishes. Each dish contained 50 mL of Murashige and Skoog medium. Seeds for all 105 lines were sown within 2 days to minimize age variation. The experiment comprised 1008 RIL individual plants in total, including eight replicates for each RIL and each parental line. Growth experiments were performed from September to June, representing the winter annual life cycle of both lines (Baskin and Baskin 1972). After 3 days at 4 °C, all petri dishes were placed in three incubators (GROWTH CABINET- HNM - S11, Koito Industries, Ltd, Japan) with a 12-h photoperiod, day/night temperatures of 20/20 °C for 18 days. Seedlings were randomly chosen and were transferred to 10-cm diameter clay pots with the same soil combination as in the SSE. All potted plants were transferred to the outside experimental garden within 2 days to reduce effects of environmental differences on growth. Plants were then grown under the natural seasonal environment. The garden was fenced to exclude mammalian herbivores. Pots were distributed with a randomized block design across (24 1.2 m × 1.2 m) wooden blocks filled with decomposed granite soil. Plants were watered four times a week except on rainy days. Phenological traits were recorded almost every day until all vegetative and reproductive parts were completely withered. Traits analysed for QTL study were the time of bolting, the time of flowering initiation and the time of flowering termination (last flower senescence) **[see****Supporting Information—Dataset Table 3****]**. These are the traits that showed big contrast among parental lines (Cvi and Ler) in our sequential seeing experiment. In addition, the variation of flowering period among cohorts in SSE was the key factor for the senescence synchrony hypothesis; i.e. desynchrony in flowering time among cohorts and the synchrony for senescence are causing the variation in flowering period. We performed QTL analysis for flowering period as well but because the exact same loci as flowering initiation were detected, we are not showing the data for flowering period. Overall these traits are all connected and shaping the senescence synchrony.

### QTL analysis

To map QTL using the RIL population, a set of 144 markers covering most of the arabidopsis genetic map was used from the previously published RIL Ler/Cvi map (Alonso-Blanco *et al.* 1998). Genotype and phenotype data (mean of eight replicates for each line) were merged and saved as .csv files. Quantitative trait locus analysis was performed with simple and composite interval mapping (Arends *et al.* 2010) applying a Haley–Knott (HK) regression method (Haley and Knott 1992), using the EM algorithm, Haldane map function and a window size of 2-cM interval, for all the measured traits, using the R/QTL library in the R environment (Broman *et al.* 2003). To determine an appropriate significance threshold for each QTL, empirical thresholds for significance (*P* < 0.05) and suggestive (*P* < 0.63) LOD scores were determined using 10 000 permutations (Churchill and Doerge 1996; Broman *et al.* 2006). Quantitative trait locus positions were assigned to relevant regions at the point of the maximum likelihood odds ratio (LOD). A stepwise regression method was used for the genome scan to identify the most significant markers. Confidence intervals (95 %) around each significant QTL peak were established using the *baysint* function. Additional information regarding the extent of variation explained by each QTL, as well as the effect associated with each parental allele, was gathered using the *sim.geno*, *makeqtl*, *fitqtl*, *effectplot* and *effectscan* functions in R/QTL library. The estimated Additive effect and Epistatic interactions between QTL and the percentage of variance explained by each QTL, as well as the total variance explained by all of the QTL affecting a trait were identified using the *scantwo* (pairscan) function. The Bayesian method was used to determine the 95 % CI (Broman and Sen 2009). Briefly, the interval is obtained by assuming 10^LOD is the true likelihood function, assuming *a priori* that the QTL is equally likely to reside anywhere on the chromosome. The posterior density can be derived from these two assumptions. The Bayes credible interval is defined as the interval for which the posterior exceeds a given probability, in this case 0.95. Original chromosomal position and proportion of phenotypic variance (*R*^2^) were explained by each QTL.

## Results

### Sequential seeding experiment

#### Flowering initiation was desynchronized in all accessions within each group.

**Figure 2.**
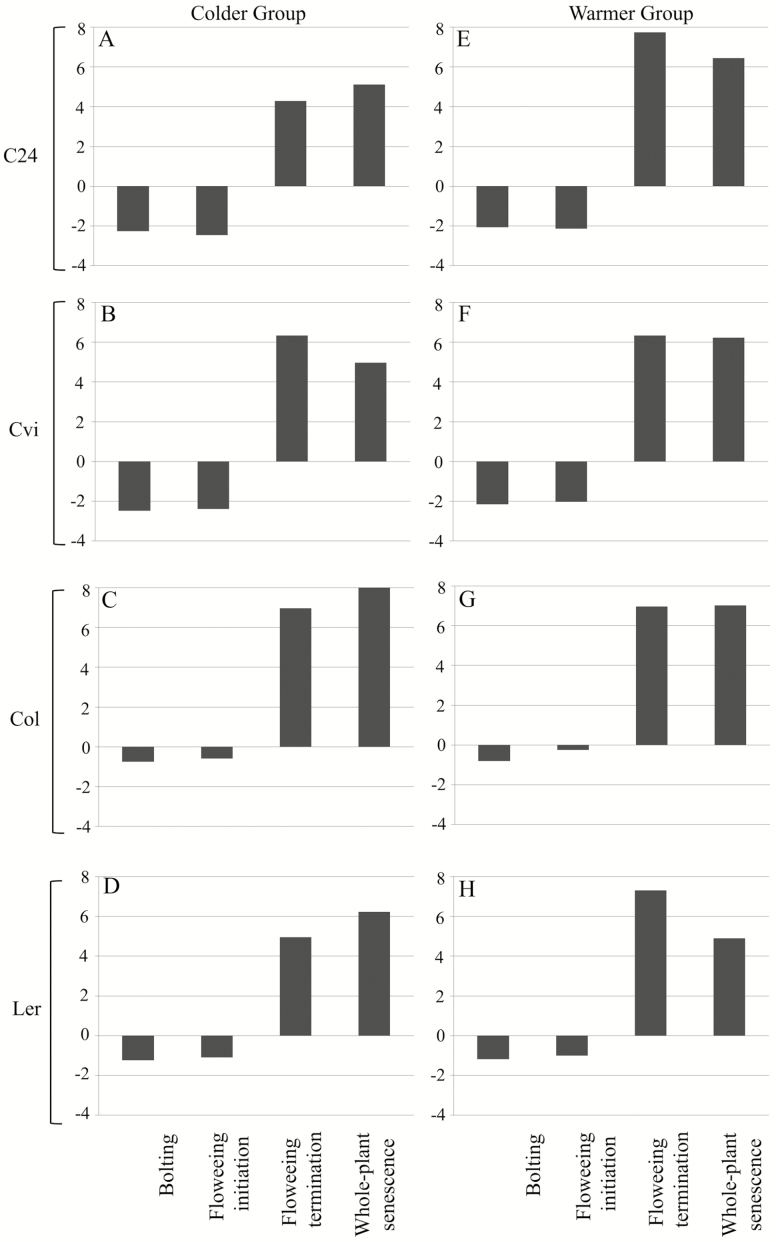
SIg values of bolting, flowering initiation, flowering termination and whole-plant senescence are presented for (A) C24, (B) Cvi-0, (C) Col-0 and (D) Ler-1 in Colder Group and for (E) C24, (F) Cvi-0, (G) Col-0 and (H) Ler-1 in Warmer Group.

A series of SSE using four accessions of arabidopsis (i.e. C24, Cvi-0, Col-0 and Ler-1), all known as early-flowering accessions (Donohue 2002), was conducted. Unlike late-flowering accessions of arabidopsis, even without vernalization, these accessions flower early (Wilczek *et al.* 2009). The timing of bolting, flowering initiation, flowering termination and whole-plant senescence were measured. In all accessions, desynchronization (SIg < 0)—please see ‘Materials and Methods’—was detected for bolting and flowering initiation and strong synchrony (SIg > 4) was detected for flowering termination and whole-plant senescence (Fig. 2). Synchrony in phenological traits, i.e. a positive synchronization index (SIg > 0), is expected if the trait is being regulated in environmental-dependent manner. As expected, SIg values for bolting and flowering initiation indicated that variance across the five cohorts became 2–5 times larger than for germination timing. In both groups, plants responded to their own environmental conditions. Cohorts that germinated first flowered before the cold period, but later cohorts flowered at the onset of warmer times, which desynchronized bolting and flowering initiation (Fig. 2).

#### Senescence was synchronized among cohorts for each accession within each group.

All accessions indicated strong synchrony of flowering termination and whole-plant senescence (Sig > 0; Fig. 2). SIg values for flowering termination and whole-plant senescence indicated that variances across cohorts for C24, Cvi-0, Col-0 and Ler-1, respectively, became ~1/20, 1/100, 1/125 and 1/30, of the variance of germination timing in Colder Group and 1/250, 1/100, 1/125 and 1/150, of the variance of germination timing in Warmer Group (Fig. 2). For whole-plant senescence, it was 1/50, 1/30, 1/500 and 1/100 in group I and 1/100, 1/100, 1/150 and 1/30 in Warmer Group. Synchrony of these traits was observed despite desynchrony in flowering initiation. Overall, seasonally dependent regulation of timing was suggested for flowering termination and whole-plant senescence in all accessions, which is consistent with our previous results (Stinchcombe *et al.* 2004).

### Plants flowered after constant PTU

**Figure 3.**
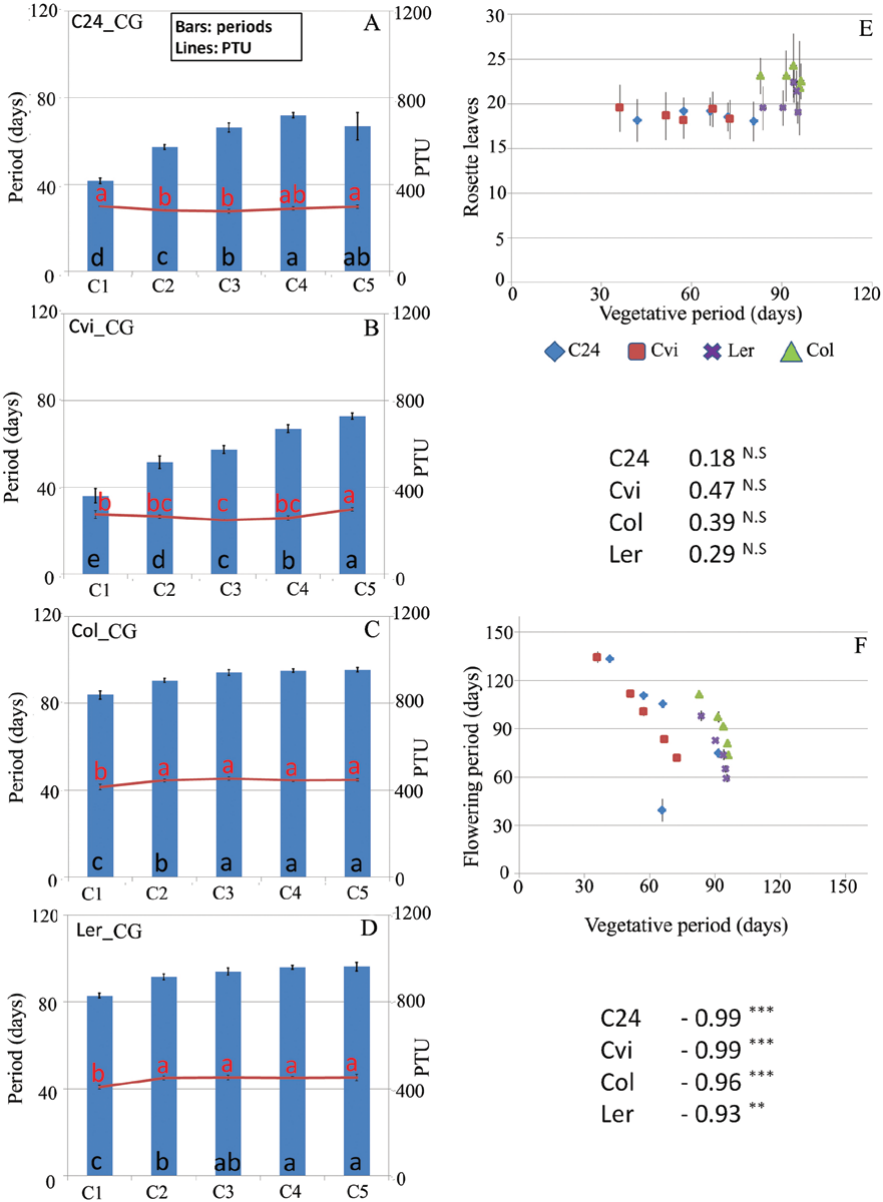
Comparison of the vegetative periods and PTU across cohorts in the four accessions and leaf number at bolting and flowering period compared to the vegetative period. Duration of vegetative periods (blue bars) and PTU values during the corresponding periods (red lines) are presented for five cohorts of (A) C24, (B) Cvi-0, (C) Col-0 and (D) Ler-1 in Colder Group. (E) The dependency of the rosette leaf number at bolting during the vegetative period, and (F) the relationship between vegetative and flowering periods. The duration of vegetative periods was calculated as the number of days from germination to flower initiation. In (A–D), means and standard deviations (SDs) are presented. Different letters at the bottoms of the bars and next to the lines indicate significant differences (*P* < 0.05) in periods and PTU values between cohorts. In (E) and (F), cohort means are plotted with different symbols for four accessions. Correlation coefficients (*r*) are also listed for each accessions (***, **, N.S.; *P* < 0.001, *P* < 0.01, no significance at *P* < 0.05, respectively). Standard deviations for the number of rosette leaves are represented by vertical bars (E). The same information for Warmer Group is shown in **Supporting Information—Fig. S1**. CG: Colder Group.

In the first 24 days after transferring the plants to the greenhouses, they were warmed (20 °C for Colder Group and 23 °C for Warmer Group; **see****Supporting Information—Dataset Table 2**) to mimic a short, warm autumn. Because each cohort was transferred 1 week after the previous one, this warm autumn lasted 24 days for C1, 17 days for C2, 13 days for C3, 8 days for C4 and only 2 days for C5. In addition, different temperature conditions for each group caused the timing of life-cycle events to differ. ‘Warmer Group’ was manually caused to experience about 3 °C warmer temperatures throughout the whole experiment compared to ‘Colder Group’. This caused plants in ‘Colder Group’ to spend longer in the flowering phase before senescing (Fig. 1). Photothermal unit-dependent regulation of flowering time (flowering after passing a certain threshold of accumulated PTU) has been studied in the phenology model of arabidopsis (Wilczek *et al.* 2009). In the present study, later cohorts started growth on days with fewer PTUs than earlier cohorts, because of lower temperatures (Fig. 1). To examine whether desynchrony of bolting and flowering initiation—and therefore synchronization of senescence—are explained by PTU-dependent regulation, the vegetative PTU (PTU from the time of germination to flowering initiation) for all cohorts of each accession in both groups was calculated (Fig. 3A–D; **see****Supporting Information—Fig. S1A****–****D**). Constant vegetative PTU values were observed across the five cohorts, suggesting that earlier cohorts of these accessions flowered even before the cold regime started, after producing a constant number of leaves and when they perceived a constant level of PTU. Vegetative periods became longer from C1 to C5 in both groups, but longer vegetative periods did not result in higher vegetative PTU values (Fig. 3A–D; **see****Supporting Information—Fig. S1A–D**); and therefore, longer vegetative periods did not result in higher numbers of rosette leaves and bigger sized plants. Instead, the number of rosette leaves at bolting was constant across cohorts (Fig. 3E; **see****Supporting Information—Fig. S1E**) (*r* = 0.18, 0.47, 0.39, 0.29 and *r* = 0.11, 0.39, 0.66 and 0.10 for C24, Cvi-0, Col-0, Ler-1, in Colder Group and Warmer Group, respectively; Fig. 3E; **see****Supporting Information—Fig. S1E**). This suggested that older cohorts managed to produce a constant number of leaves after perceiving a constant amount of PTU and they flowered even before the cold regime. Flowering periods were shorter in later cohorts **[see****Supporting Information—Fig. S2****]** because flowering termination was strongly synchronized for all accessions. This resulted in a negative relationship between vegetative and flowering periods (*r* = −0.99, 0.99, 0.96, 0.93 and *r* = −0.98, 0.99, 0.85 and 0.97 for C24, Cvi-0, Col-0, Ler-1, in Colder Group and Warmer Group, respectively; Fig. 3F; **see****Supporting Information—Fig. S1F**). Rosette sizes at bolting and fruit production were more or less constant among all cohorts within each group, and could not be explained simply by either vegetative/flowering period or PTU **[see****Supporting Information—Fig. S3****]**. This is expected for rosette leaves since all cohorts received a similar number of PTU during the vegetative period, but for fruit numbers, one may assume that a plant that received more PTU during the flowering period would make more fruits, but this was not the case in our previous study either. According to some previous studies, the limitation in growth and fruit production may occur due to loss of meristem activity after longer vegetative periods (Kudoh *et al.* 1996; Kudoh *et al.* 2002; Miryeganeh *et al.* 2018).

### Analysis of QTL

Genetic architecture of studied traits was addressed with a QTL approach using the R/QTL library in the R environment (Arends *et al.* 2010). Parental lines were contrasted for reproductive traits according to the SSE. The Cvi-0 accession in both groups was characterized by earlier flowering and earlier senescence compared with Ler-1 (Fig. 1). Data for all detected QTL are shown in **Supporting Information—Dataset Table 1**, including the original chromosomal position, nearest marker, genetic position (cM), physical position (Mbp), LOD score, the closest flowering or senescence gene, physical position (Mbp) of the gene and proportion of phenotypic variance (*R*^2^) explained by each QTL. The sum of significant (*P* < 0.05) and suggestive QTL for each trait varied from one to three and each QTL explained 10–28 % of the phenotypic variance. Overall, the composite interval mapping uncovered six unique QTL for the three reproductive traits in the RILs. Two significant QTL were detected for flowering initiation (one QTL each on chromosome 1 and 5). Only suggestive QTL were detected for bolting time and flowering termination (senescence) timing **[see****Supporting Information—Table S1****]**. *fitqtl* was used to fit a multiple-QTL model and to estimate the percent phenotypic variance explained by each QTL. The QTL region on the upper arm of chromosome 1 appeared as the major locus responsible for observed variation in flowering time trait with the highest LOD score **[see****Supporting Information—Fig. S5]**. The *c1.loc6* locus accounted for the majority of the phenotypic variation for flowering time (*R*^2^ = 27.77 %). On position 66 cM of chromosome 4, and position 67 of chromosome 5, QTL were found for flowering termination, indicating that these loci might be involved in senescence timing but we cannot be certain since they are only suggestive QTL. In the present study, all detected significant and suggestive QTL had LOD scores equal to or greater than 2.4 **[see****Supporting Information—Table S1****]**. The sum of the phenotypic variance explained; i.e. the heritability estimate of the trait by all significant and suggestive QTL was 10.05 % for bolting time, 48.99 % for flowering initiation time, 21.59 % for flowering termination time **[see****Supporting Information—Table S1****]**. In other words, detected QTL for these traits could explain 10–50 % of their variation.

**Figure 4.**
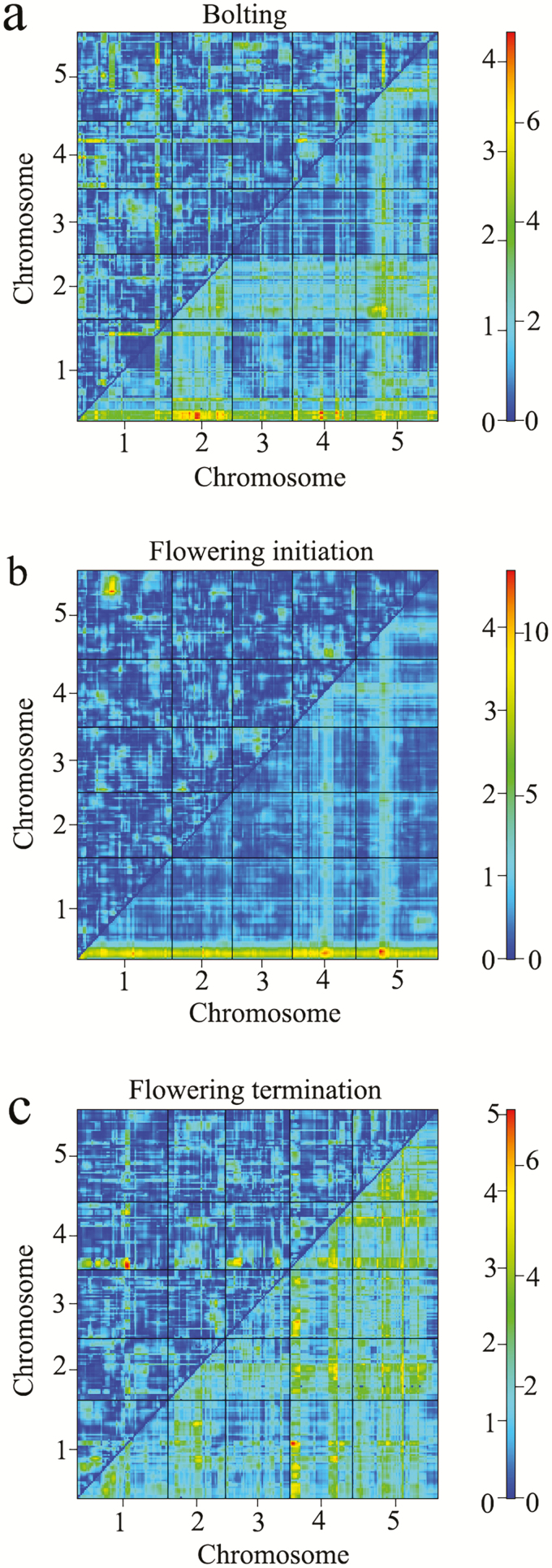
A *Scantwo* analysis of bolting period (A), flowering initiation time (B) and flowering termination time (C). The top triangle shows epistasis, while the bottom triangle shows additive interactions. The colour scale indicates LOD scores for epistasis (left) and additive interactions (right).

In all of the studied traits, although several additional QTL peaks barely crossed the significance threshold on their own **[see****Supporting Information—Fig. S5****]**, a *scantwo* analysis revealed strong additive interactions for every pair of regions in each case; i.e. ‘Chr. 1 and Chr. 2’ and ‘Chr. 1 and Chr. 4’ for bolting period, ‘Chr. 4 and Chr. 5’ for flowering initiation time, acted in an additive manner and in the case of senescence timing ‘Chr. 1 and Chr. 4’ showed an Epistatic interaction effect (Fig. 4). In addition, as two detected QTL for flowering termination time (chromosomes 4 and 5) differed in their magnitude, with the chromosome 5’s locus being the major QTL **[see****Supporting Information—Fig. S5****]**, the *scantwo* result showed that Chr. 1 also interacts with Chr. 4, which was not detected with the main scan in a single-QTL model (Fig. 4). Regions where QTL co-localized with genes involved in flowering time and leaf senescence processes are shown in **Supporting Information—Fig. S4**, and are also listed in **Supporting Information—Table S1** (please also see **Supporting Information—Note**). Several overlaps were observed between detected significant/suggestive QTL and genes known from the literature to be involved in flowering and senescence time, implying that they might be responsible for the phenotypic variation observed. The fine mapping of these loci may help to reveal their roles in reproductive traits.

## Discussion

Timing of reproduction is very important for plants, especially when they are living in a seasonal environment. During their life cycles, plants are exposed to various combinations of environmental conditions that can induce or delay reproduction. It is critical for growth, reproduction and survival to adjust and synchronize developmental events with favourable seasonal conditions (Wilczek *et al.* 2010). In fact, it has been argued that what is often termed ‘reproductive synchrony’ may simply be a consequence of individual plants selecting the same favourable time for reproduction in relation to climate (Ims 1990). Among reproductive behaviours, senescence represents the final developmental stage of the organs, and helps plants adapt to adverse environmental conditions. In monocarpic plants, reproductive senescence may occur to support yield quality (Schippers 2015). In our previous study, a novel definition and quantification method to evaluate reproductive synchrony was introduced by engaging life-history plasticity in phenological synchrony (Miryeganeh *et al.* 2018). We found senescence synchrony among plants of different ages grown in a natural environment in accessions that indicated synchrony (Lov-5 and Tamm-2) or desynchrony (C24 and Ler-1) of bolting and flowering initiation, suggesting that the timing of flowering termination and whole-plant senescence is under season-dependent regulation. We suggested that the timing of flowering is due to receiving a certain number of PTU. In that study we developed a synchrony index that can be used in SSE experiments to investigate the relationship between environmental synchrony or desynchrony of phenological events. In the present study, using two sets of environmental conditions, the greenhouse SSE demonstrated that all accessions synchronized reproductive senescence in response to their environments, by adjusting life-history traits, especially flowering period. In other words, flowering termination and whole-plant senescence synchronized among cohorts within each group (each greenhouse) and according to their own environments. In each group, timing of flowering initiation in later cohorts happened later in the season, and the later the plants flowered, the shorter their flowering period became, suggesting that plants synchronize senescence timing with the season by adjusting their flowering period. In Colder Group, where plants experienced lower temperature, the flowering period lasted longer, and flowering senescence occurred later compared to Warmer Group (Fig. 1).

The fact that all five cohorts within each group synchronized senescence and not with counterparts in the other seasonal group supports the hypothesis that seasonal conditions have a greater impact on senescence timing than age, and it shows that plants control senescence timing by adjusting the timing of life-history events based on the environment in which they are growing. This could result from an experimentally manipulated seasonal environment, and results of the previous study under natural conditions suggest that the common dictum in life-history biology, which says there is a fixed schedule for reproductive stages, might not always hold, and that it can be changed according to seasonal conditions. Although senescence occurs in an age-dependent manner in plants, except perhaps in extreme cases, plants do not die before they reproduce, even if that necessitates shortening the reproductive period and producing fruits and seeds shortly after flowering. In fact, plants are unusual organisms in that, they can set their own lifespans according to environmental conditions (Thomas 2013).

In this study we focused on temperature and light (together in forms of PTU) as two major environmental factors. We know that plants also can communicate through the volatile, senescence-inducing plant hormone ethylene. One might argue, it is possible for the oldest cohorts to induce senescence in the younger cohorts by means of this hormone. Even though this has not been tested or controlled in our study, we are confident this could not be the reason of senescence synchrony we observed among cohorts in each greenhouse. The synchronized senescence of Cvi-0 occurred ~1 month and 20 days earlier than that of Ler-1 in ‘Colder Group’ and ‘Warmer Group’, respectively, and therefore, Cvi-0 terminated flowering when Ler-1 was actively producing flowers (Fig. 1; **see****Supporting Information—Dataset Table 1**). We also had placed two replicates of a late-flowering arabidopsis accession (Tamm-2) as control in each greenhouse—with same age as cohort 5 of other accessions—and these two Tamm-2 plants senesced even another month later than Ler-1 (data not shown; this is consistent with our previous SSE (Miryeganeh *et al.* 2018)). Therefore, we concluded that the observed senescence was controlled internally by the plants in response to temperature and light as measured seasonal environment factors in this study.

Shortening the flowering period in later cohorts enables plants to reproduce before the hot season and can preclude reproduction. A similar strategy can be seen in crop breeding and drought escape, which enables plants to complete their life cycles prior to the onset of drought, thus avoiding moisture limitations in the hot season (Fletcher *et al.* 2015). Another example is that spring-flowering, Mediterranean-adapted plants (e.g. barley, wheat) often accelerate development in response to lengthening days, which allows them to complete their life cycles before hot, dry conditions of summer. In tropical plants, such as sorghum, shortening days of late summer signal the end of summer and the onset of the autumn monsoon rains, which are favourable for grain filling (Dingkuhn *et al.* 2008). There is also a close association between senescence and induction of seed maturation in cereal crops (Kohl *et al.* 2012; Hollmann *et al.* 2014). The stay-green trait delays leaf senescence in maize (Antonietta *et al.* 2014), but when plants must compete for resources, this trait is actually undesirable, and stay-green maize lines cannot outcompete early-senescing lines when grown at high density (Antonietta *et al.* 2014). Therefore, perfect senescence timing is essential for plant productivity, and it represents an important evolutionary trait that enables plants to adapt to seasonal environments. It is safe to say that onset and progress of senescence are sensitive to seasonal change (Thomas and Ougham 2014), and the hypothesis that says synchrony among plants arises incidentally because they experience similar weather is the most parsimonious explanation for variability in reproduction (Kelly and Sork 2002). It should be noted that, in the greenhouse experiment, light could not be controlled, because plants were exposed to natural sunlight passing through the glass walls of the greenhouses. Day length, as well as temperature, is an important cue for appropriate timing of flowering and fruiting (Thomas 2013), because for most plants, declining photoperiod signals the end of the growing season. Future experiments that control photoperiod more accurately, along with temperature, may give us a more nuanced understanding of the interplay between these factors. For instance, bud-set timing is more influenced by diminishing day length than by low temperatures (Bohlenius *et al.* 2006; Savolainen *et al.* 2007). Although this study attempted to increase our understanding of ‘senescence synchrony’ introduced in the previous work (Miryeganeh *et al.* 2018), there are still many seasonal/environmental factors that can be controlled and tested in order to investigate further the concept of reproductive synchrony and/or desynchrony and to possibly expand this phenomenon to other plants species. Future studies will reveal novel and exciting results in this field to provide further insights into the complex regulatory network of senescence synchronization in plants. We hope that drawing attention to this direction will stimulate future research in this area. Phenological studies performed in different ecological settings by manipulating the temporal pattern of seasonal conditions and subsequently comparing reproductive synchrony of individuals would be valuable to advance our understanding of the ecology and evolution of senescence synchrony and the mechanism behind it. It is important to identify processes that trigger senescence synchrony in response to seasonal environmental and to investigate mechanisms behind it to see whether regulation of senescence (genetically and/or epigenetically) is conserved in other plant species.

Overall, to understand the basis of senescence timing synchrony, it is necessary to explore its underlying mechanisms. Thus, genetic knowledge of the basis of synchronous reproductive traits and their natural variability, the effect of seasonal conditions on expression of these traits and their fitness consequences are needed. Whether senescence is a synchronized process in other species and whether levels of synchrony depend on competition or environmental cues are unknown.

Ler/Cvi RILs in the QTL mapping was used, in hopes of identifying loci that control reproductive traits and adjustment of flowering period or even senescence synchrony. Genetics of flowering time in arabidopsis have been examined extensively, and molecular pathways have been well-characterized by a variety of means, including QTL mapping. However, almost all QTL studies employed the laboratory strain, Col, as a mapping parent, and were performed under controlled laboratory conditions (Juenger *et al.* 2005; Werner *et al.* 2005; Shindo *et al.* 2007; Simon *et al.* 2008; Balasubramanian *et al.* 2009; Brachi *et al.* 2010; Strange *et al.* 2011). Many genes control flowering time, but few have been shown to be associated with natural flowering time variation. Some QTL that do not overlap with candidate reproductive genes were identified; however, some flowering or senescence genes are located at close to, but not at the exact location as these QTL **[see****Supporting Information—Fig. S4**; **Supporting Information—Table S1****]**. For example, AXR-1 was detected as the nearest marker for QTL *c1.loc6* on Chr. 1, for flowering initiation, but has not been described in previous QTL studies of flowering time. Finding novel loci involved in flowering time is rare, but has also been demonstrated in other studies that mapped natural populations in the field (Brachi *et al.* 2010; Grillo *et al.* 2013). It should also be noted that experiments involving different environmental conditions might identify different sets of QTL, since development (Pathak and Zhu 2007) is epigenetic and involves interactions among direct and indirect control factors. These interactions may vary during ontogeny (Atchley 1990; Chakraborty and Zhao Bang 2011). Thus, we expect that different QTL might be detected in studies of reproductive traits that are done in different laboratory or environmental conditions. Different QTL in studies using other lines may yet show that genetic control of reproductive traits varies between lines and natural populations.

Three significant and/or suggestive QTL: c1.loc6, c4.loc44 and *c5.loc36* for flowering initiation time were identified on Chr. 1, Chr. 4 and Chr. 5, respectively. *C1.loc6* overlaps with the AXR1 gene, which has not been reported for flowering time before. *C5.loc36* overlaps with the flowering gene, FPF1, and *c4.loc44* was co-localized with the senescence gene, NYC1, which encodes a chlorophyll b reductase involved in degradation of chlorophyll b and LHCII (light-harvesting complex II). It is annotated as a gene associated with leaf senescence (Nakajima *et al.* 2012). NYC1 also participates in seed maturation (Zhang and Gan 2012). Surprisingly, this locus is associated with flowering initiation time, but not with flowering termination, suggesting that other genes are responsible for the phenotypic variation.

Two suggestive QTL, *c4.loc58* and *c5.loc36*, were detected for flowering termination and overlapped with WRKY53 and ANAC092, respectively. The transcription factor for the senescence regulatory gene, WRKY53, is involved in the progression of leaf senescence (Guo and Gan 2006). ANAC092 encodes an NAC family transcription factor, which is a key factor in regulation of leaf senescence (Gepstein *et al.* 2003).

The regulatory gene, FRI, has received the most attention as a determinant of flowering time in natural arabidopsis populations (Clarke and Dean 1994; Johanson *et al.* 2000; Le Corre *et al.* 2002; Simpson and Dean 2002). Functional FRI alleles result in accumulation of FLC mRNA, which inhibits flowering. Vernalization reduces the sensitivity of FLC to FRI, and thereby promotes flowering. Several studies support the role of FRI in controlling natural flowering time variation (Le Corre *et al.* 2002; Parkin *et al.* 2005; Korves *et al.* 2007). Despite this substantial body of research demonstrating the contribution of FRI to flowering time variation, the confidence interval determined for the QTL peak on Chr. 4 on *main scan* does not exactly cover the FRI position, which is located on top of Chr. 4: http://www.arabidopsis.org/servlets/TairObject?type=geneandid=129636 (almost 10 cM apart; **see****Supporting Information—Table S1**). Moreover, the QTL effect was not found to be significant which is not surprising since none of the parents (Ler and Cvi) has an active FRI.

Although in the *main scan* of the ‘flowering initiation time’ trait, an additional QTL peak on Chr. 4 barely crossed the significance threshold on its own **[see****Supporting Information—Fig. S5****]**. A *scantwo* analysis revealed strong additive interactions for Chr. 4 with the Chr. 1 QTL (Fig. 4). Another additional QTL peak that proved barely significant was the Chr. 5 QTL, which also appeared to have a strong additive interaction with Chr. 1 upon *scantwo* analysis (Fig. 4). This demonstrates that the QTL on Chr. 1 has significant phenotypic effects, and acts with Chr. 4 and Chr. 5 in an additive manner. The top of arabidopsis Chr. 5 (Putterill *et al.* 1995) contains the well-characterized flowering time genes CO (Simpson *et al.* 2003), FY (Michaels and Amasino 1999) and FLC (Atwell *et al.* 2010), among others. It is possible that these candidate genes are causal genetic elements underlying QTL; however, functional experiments are required to confirm their role in reproductive timing variation.

Consistent with the SSE, senescence synchrony among all RIL plants and very slight ensuing variation in senescence timing among lines was found. This resulted in weak QTL detection for this trait. Perhaps genetic architecture of small-effect QTL is favoured in synchronized behaviours as a means of ensuring synchronous senescence among plants under suitable seasonal conditions. Quantitative trait locus detected in flowering initiation, on the other hand, had greater effects than QTL detected in flowering senescence. Synchronous flowering in selfing species such as arabidopsis is not required and is more likely to tolerate large QTL effects on flowering. In order to synchronize senescence, plants had to desynchronize flowering. Therefore, we predict that QTL for flowering time could be associated with senescence synchrony as well. In the *mainscan* for senescence timing QTL peaks on Chr. 4 and Chr. 5 that almost reach the significance threshold was found **[see****Supporting Information—Fig. S5****]** and a *scantwo* analysis later revealed strong interaction between Chr. 4 and Chr. 1 QTL, which is similar to flowering QTL (Fig. 4). This might suggest that the genes responsible for senescence synchrony are those that also desynchronize flowering by adjusting flowering phase length.

Unfortunately QTL mapping typically produces large genetic intervals and contains tens to hundreds of genes which make it difficult to determine the best candidates for the causal genes. For example, detected QTL might be detected as a single QTL, when two loci are closely linked or a large QTL can be divided into multiple small variation QTL. Therefore, further analysis such as fine mapping, advanced intercross RILs (AI-RILs) and methods that help prioritize QTL candidate genes using a computational approach would be very helpful in unravelling genotype-to-phenotype relationships (Luquez *et al.* 2006; Balasubramanian 2009). To prioritize QTL candidate genes, one approach consists of using genes previously identified as influencing traits under study to test whether these explain a QTL (Chen *et al.* 2012). Developing near isogenic lines (NILs) can separate the many alleles within these QTL and their impacts on senescence and flowering time may be unequivocally estimated. Additionally, these NILs could be grown under diverse seasonal conditions to understand environmental influences on these traits and their interaction with the underlying genetics.

## Supporting Information

The following additional information is available in the online version of this article—

**Table S1.** Original chromosomal position, nearest marker, genetic position (cM), physical position (Mbp), LOD score, the closest flowering or senescence gene name, physical position (Mbp) of the gene and its Atg number explained by each quantitative trait locus (QTL). Significant (*P* < 0.05) and suggestive (*P* < 0.63) LOD scores for each QTL are shown with ** and *, respectively.

**Figure S1.** Comparison of the vegetative periods and photothermal unit (PTU) across cohorts in the four accessions and the leaf number at bolting and flowering period compared to the vegetative period.

**Figure S2.** The duration of the flowering periods (red bars) and photothermal unit (PTU) values during the corresponding periods (blue lines) are presented for five cohorts of (A) C24, (B) Cvi-0, (C) Col-0 and (D) Ler-1 in Colder Group (CG) and for (E) C24, (F) Cvi-0, (G) Col-0 and (H) Ler-1 in Warmer Group (WG).

**Figure S3.** The dependency of the rosette diameter at bolting on the vegetative period and photothermal unit (PTU) for Colder Group (A, B) and Warmer Group (E, F), dependency of fruit production on the on the flowering period and PTU for Colder Group (C, D) and Warmer Group (G, H).

**Figure S4.** A schematic map of localization of significant or suggestive quantitative trait loci (QTLs) that overlap with known flowering and senescence candidate genes from the literature.

**Figure S5.** Quantitative trait locus (QTL) analysis of reproductive traits.

**Note.** Candidate genes overlapping all significant and suggestive quantitative trait loci (QTLs).

plaa018_suppl_Supplementary_Material

plaa018_suppl_Supplementary_Dataset_Tables

## Availability of Data

Data from phenological measurements for SSE, meteorology during the SSE experiments, phenological measurements for RILs and marker information are provided in **Supporting Information—Dataset Tables 1****–****3**, respectively.
