# Supplementary material for: Synchronization of senescence and desynchronization of flowering in Arabidopsis thaliana
Source: AoB Plants. 2020 May 9;12(3):plaa018. doi: 10.1093/aobpla/plaa018 (PMC7299267; doi:10.1093/aobpla/plaa018)
Supplement: plaa018_suppl_Supplementary_Material [file plaa018_suppl_supplementary_material.pdf]

Supplementary Information for

“S**ynchronisation of senescence and de-synchronisation of flowering in *Arabidopsis thaliana*”**

This file contains Supplementary Table S1, Supplementary Fig. S1, Supplementary Fig. S2, Supplementary Fig. S3, Supplementary Fig. S4 and Supplementary Fig. 5.

Supplementary Table S1. Original chromosomal position, Nearest marker, Genetic position (cM), Physical position (Mbp), LOD score, the closest flowering or senescence gene name, Physical position (Mbp), of the gene and its Atg number explained by each QTL.significant (P < 0.05) and suggestive (P < 0.63) LOD scores for each QTL are shown with ** and * respectively.

Trait	Chr.	locus	Nearest marker	Genetic position (cM)	LOD	variance explained (R2)	Physical position (Mbp)		nearest gene	Gene number
							Marker	nearest gene/s		
Bolting time	1	c1.loc2 *	PVV4	0	2.41	10.05	0.17	0.03	LHY (LATE ELONGATED HYPOCOTYL)	AT1G01060
		c1.loc10	HH.335C	2	3.32	13.54	1.19	1.19	CRY2 (CRYPTOCHROME 2)	AT1G04400
		c1.loc40	AD.121C	10	3.24	13.23	3.11	3.07	PIF3 (PHYTOCHROME INTERACTING FACTOR 3)	AT1G09530
								3.01	PHYA (PHYTOCHROME A)	AT1G09570
Flowering initiation	1	c1.loc2	PVV4	2	6.16	23.68	0.17	0.03	LHY (LATE ELONGATED HYPOCOTYL)	AT1G01060
		c1.loc6 **	AXR-1	6	7.42	27.77	1.50	1.50	AXR1 (ARABIDOPSIS AUXIN RESISTANT 1)	AT1G34180
		c1.loc8	HH.335C	8	6.58	25.06	3.11	3.07	PIF3 (PHYTOCHROME INTERACTING FACTOR 3)	AT1G09530
								3.01	PHYA (PHYTOCHROME A)	AT1G09570
	4	c4.loc2	ANL2	0	0.99	4.25	0.30	2.69	FRI (FRIGIDA)	AT4G00650
		c4.loc44 *	CH.238C	44	2.40	9.99	7.19	7.68	NYC1 (NON-YELLOW COLORING 1)	AT4G16250
		c4.loc84	BH.342C	84	0.77	3.31	18.49	17.44	RCCR (RED CHLOROPHYLL CATABOLITE REDUCTASE)	AT4G13250
	5	c5.loc2	FD.207L	0	0.86	3.70	0.25	0.40	PNY (PENNYWISE)	AT5G02030
		c5.loc36 **	GH.121L-Col	38	2.72	11.23	8.06	8.54	FPF1 (FLOWERING PROMOTING FACTOR 1)	AT5G24860
		c5.loc56	GD.239L	58	0.89	3.82	12.91	14.01	PHYC (PHYTOCHROME C)	AT5G35840
Flowering termination	4	c4.loc2	ANL2	10	1.33	5.65	0.30	2.69	FRI (FRIGIDA)	AT4G00650
		c4.loc58 *	HH.159C-Col	66	2.46	10.24	13.85	12.39	WRKY53	AT4G23810
		c4.loc84	BH.342C	85	0.77	3.32	18.49	17.44	RCCR (RED CHLOROPHYLL CATABOLITE REDUCTASE)	AT4G37000
	5	c5.loc2	FD.207L	5	1.04	4.45	0.25	0.40	PNY (POUND-FOOLISH)	AT5G02030
		c5.loc64 *	CD.116L	67	2.75	11.35	15.64	15.86	ANAC092 (ARABIDOPSIS NAC DOMAIN CONTAINING PROTEIN 92)	AT5G39610
		c5.loc90	GB.102L	88	2.23	9.32	22.39	23.25	VIN3 (VERNALIZATION INSENSITIVE 3)	AT5G57380

Fig. S1

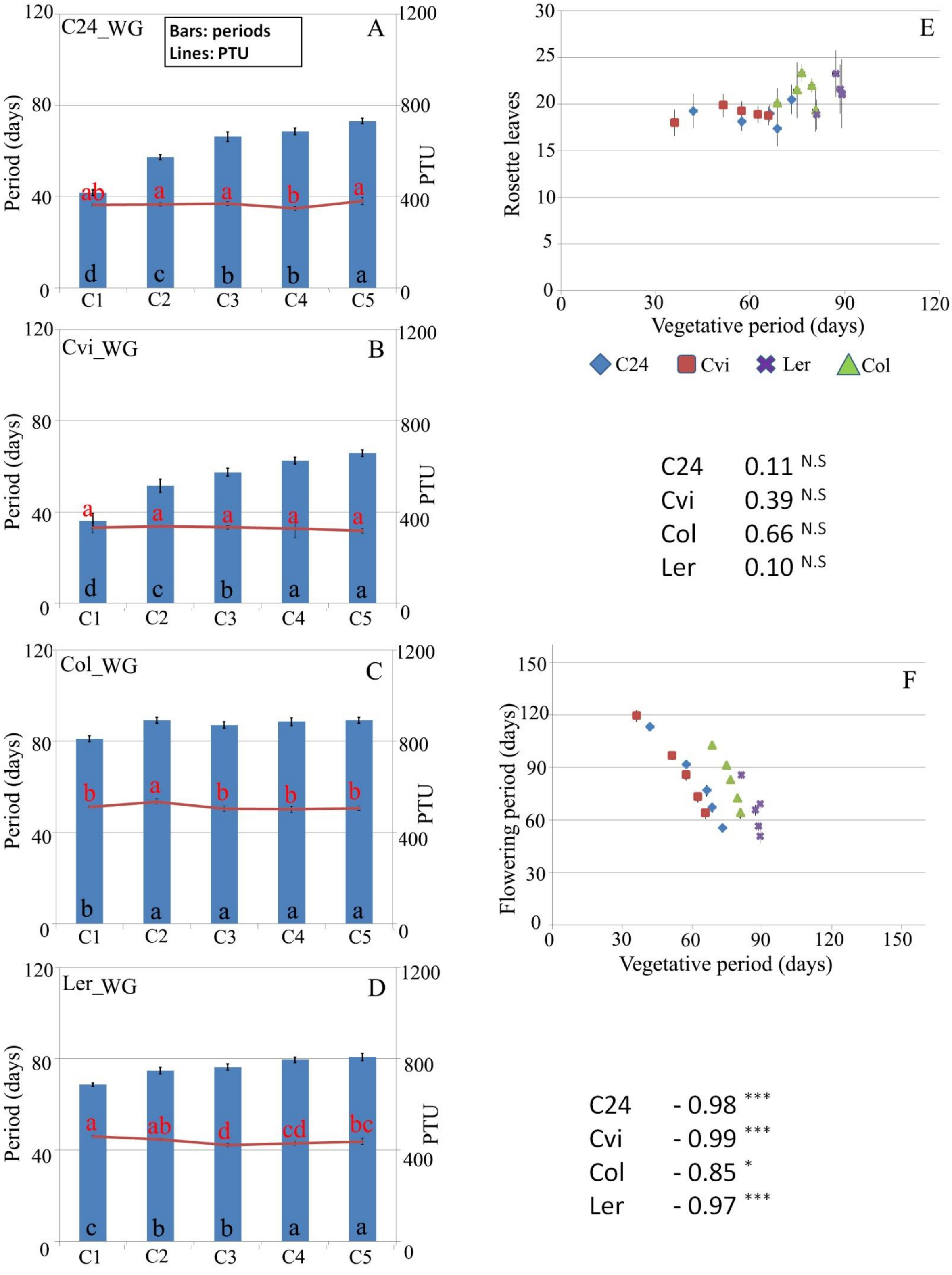

C24_WG
Bars: periods
Lines: PTU
A
Period (days)
PTU
Cvi_WG
B
Col_WG
C
Ler_WG
D
C1
C2
C3
C4
C5
E
Rosette leaves
Vegetative period (days)
C24
Cvi
Ler
Col
C24 0.11 N.S
Cvi 0.39 N.S
Col 0.66 N.S
Ler 0.10 N.S
F
Flowering period (days)
Vegetative period (days)
C24 - 0.98 ***
Cvi - 0.99 ***
Col - 0.85 *
Ler - 0.97 ***

Supplementary Fig. S1| Comparison of the vegetative periods and PTU across cohorts in the four accessions and the leaf number at bolting and flowering period compared to the vegetative period. Duration of vegetative periods (blue bars) and PTU values during the corresponding periods (red lines) are presented for five cohorts of (a) C24, (b) Cvi-0, (c) Col-0, and (d) Ler-1 in Warmer Group . (e) The dependency of the rosette leaf number at bolting on the vegetative period, and (f) the relationship between vegetative and flowering periods. The duration of the vegetative periods was calculated as the number of days from germination to flower initiation. In a-d, the means and standard deviations (SD) are presented. Different letters at the bottom of the bars and next to the lines indicate significant differences ($P < 0.01$) in periods and PTU values between cohorts. In e and f, the cohort means are plotted with different symbols for four accessions. Correlation coefficients (r) are also listed for each accessions (***, **, N.S.; $P < 0.001$, $P < 0.01$, no significance at $P < 0.01$, respectively). Standard deviations for the number of rosette leaves are represented by vertical bars (e). This figure shows the same information as Fig. 3, but for Warmer Group (WG).

Fig. S2

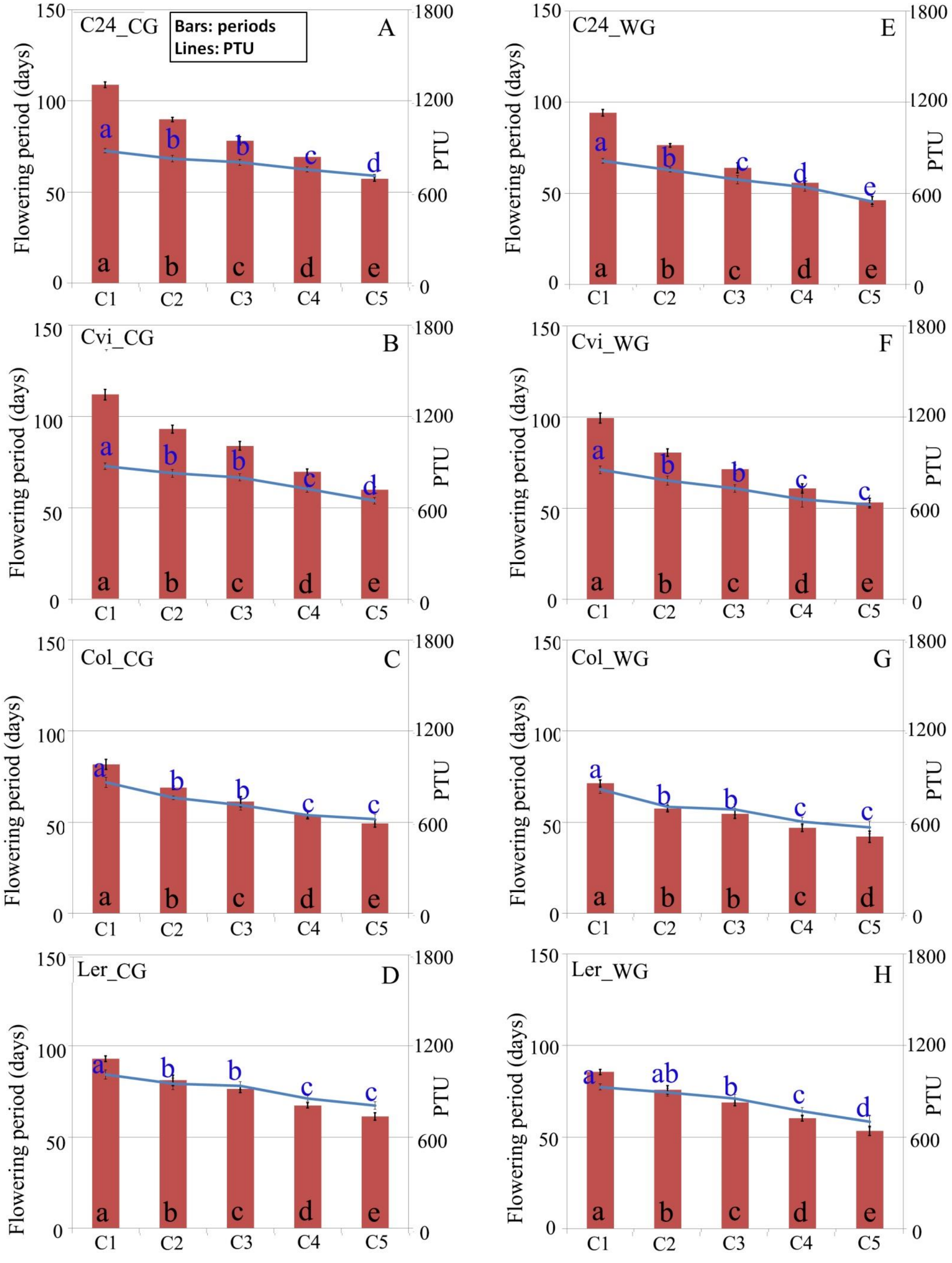
Bars: periods
Lines: PTU
C24_CG
A
Cvi_CG
B
Col_CG
C
Ler_CG
D
C24_WG
E
Cvi_WG
F
Col_WG
G
Ler_WG
H
Flowering period (days)
PTU
C1
C2
C3
C4
C5

Supplementary Fig. S2| The duration of the flowering periods (red bars) and PTU values during the corresponding periods (blue lines) are presented for five cohorts of (a) C24, (b) Cvi-0, (c) Col-0, and (d) Ler-1 in Colder Group (CG) and for (e) C24, (f) Cvi-0, (g) Col-0, and (h) Ler-1 in Warmer Group (WG). The means and standard deviations (SD) are presented. Different letters at the bottom of the bars and next to the lines indicate significant differences ($P < 0.05$) in the periods and PTU between cohorts. CG: Colder Group, WG: Warmer Group.

Fig. S3

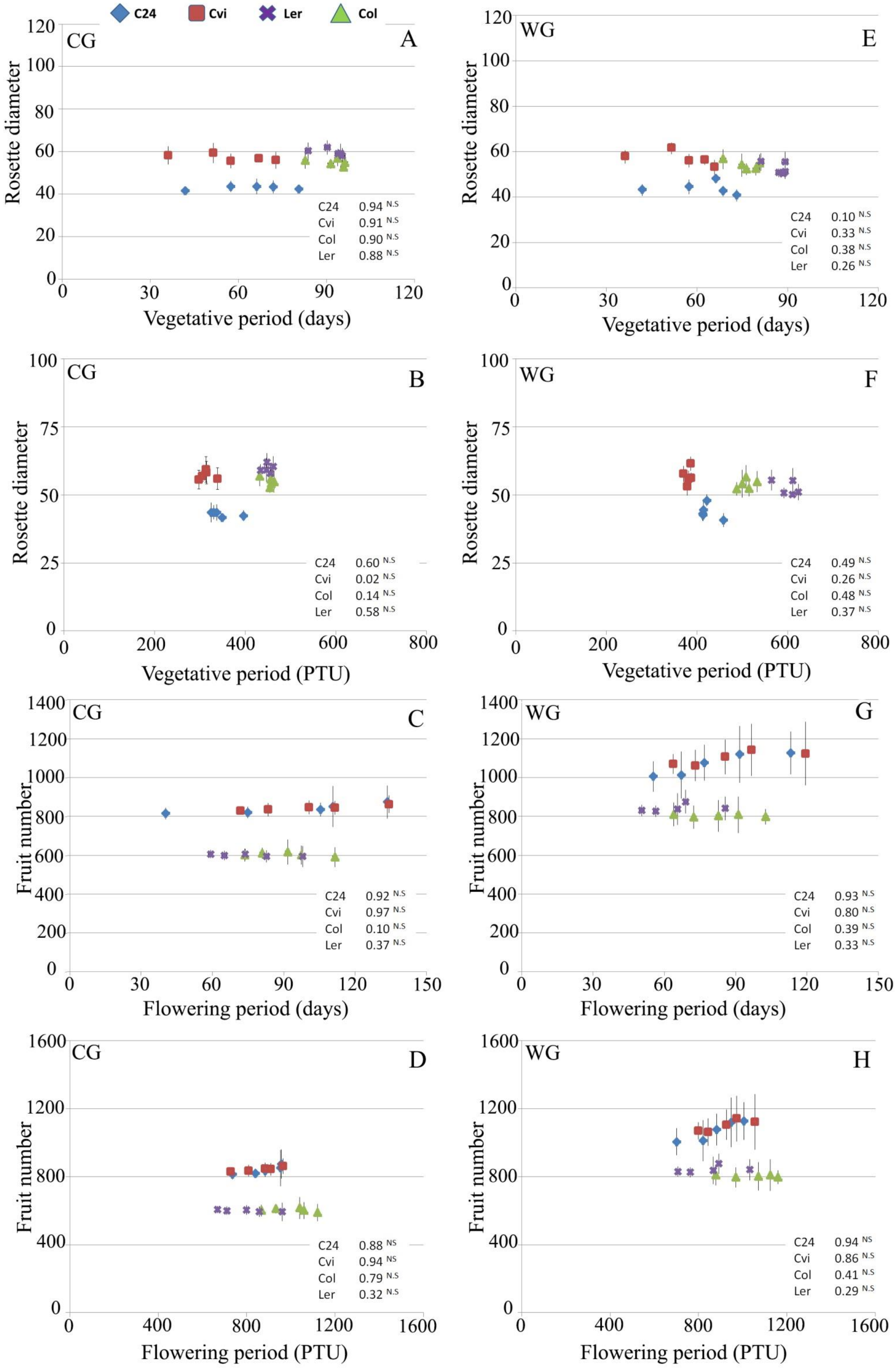
C24
Cvi
Ler
Col
CG
A
Rosette diameter
Vegetative period (days)
C24 0.94 N.S
Cvi 0.91 N.S
Col 0.90 N.S
Ler 0.88 N.S
WG
E
Rosette diameter
Vegetative period (days)
C24 0.10 N.S
Cvi 0.33 N.S
Col 0.38 N.S
Ler 0.26 N.S
CG
B
Rosette diameter
Vegetative period (PTU)
C24 0.60 N.S
Cvi 0.02 N.S
Col 0.14 N.S
Ler 0.58 N.S
WG
F
Rosette diameter
Vegetative period (PTU)
C24 0.49 N.S
Cvi 0.26 N.S
Col 0.48 N.S
Ler 0.37 N.S
CG
C
Fruit number
Flowering period (days)
C24 0.92 N.S
Cvi 0.97 N.S
Col 0.10 N.S
Ler 0.37 N.S
WG
G
Fruit number
Flowering period (days)
C24 0.93 N.S
Cvi 0.80 N.S
Col 0.39 N.S
Ler 0.33 N.S
CG
D
Fruit number
Flowering period (PTU)
C24 0.88 NS
Cvi 0.94 NS
Col 0.79 N.S
Ler 0.32 N.S
WG
H
Fruit number
Flowering period (PTU)
C24 0.94 NS
Cvi 0.86 N.S
Col 0.41 N.S
Ler 0.29 N.S

Supplementary Fig. S3| The dependency of the rosette diameter at bolting on the vegetative period and PTU for Colder Group (**a, b**) and Warmer Group (e, f), dependency of fruit production on the on the flowering period and PTU for Colder Group (**c, d**) and Warmer Group (g, h). The durations of the flowering periods were calculated as the number of days from flowering initiation to termination. The cohort means are plotted with different symbols for four accessions. The standard deviations of the rosette diameter and number of fruits are represented by vertical bars. CG: Colder Group, WG: Warmer Group.

Fig. S4

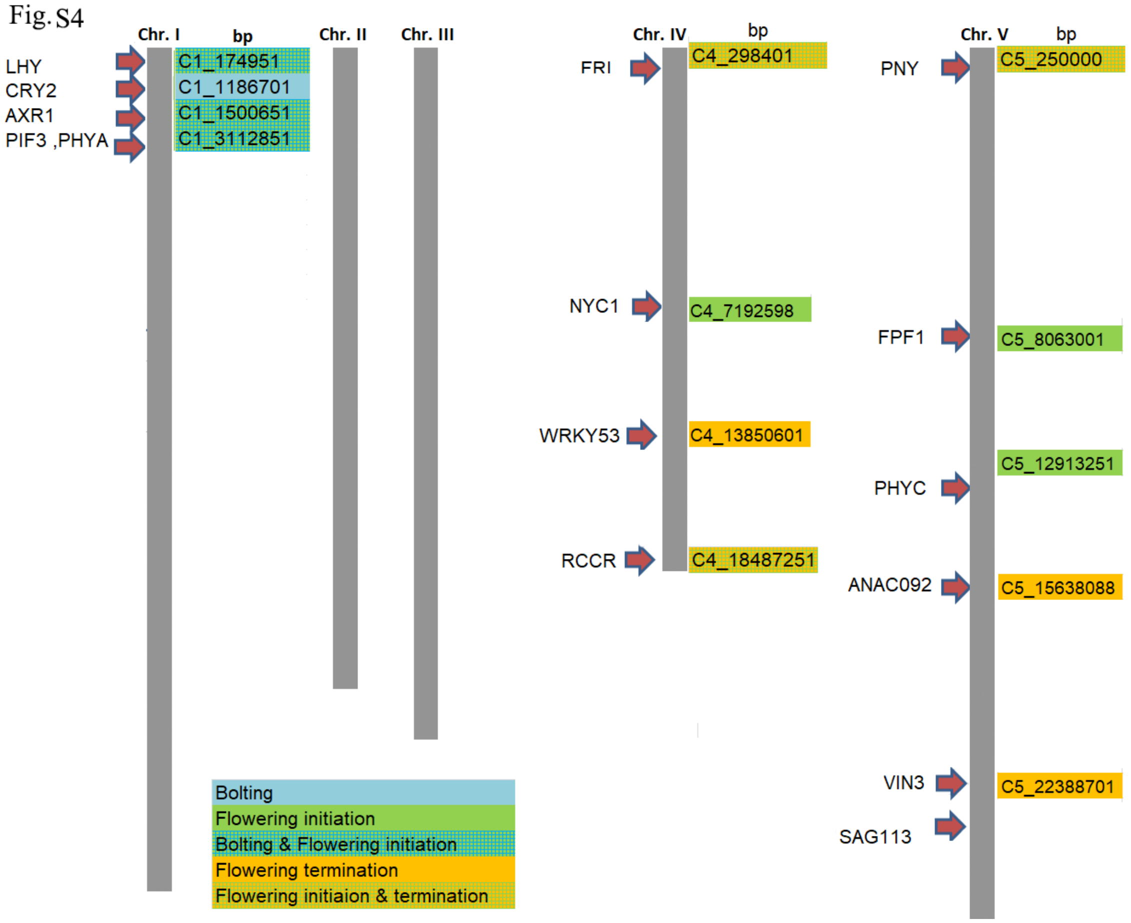

Supplementary Fig. S4| A schematic map of localization of significant or suggestive QTLs that overlap with known flowering and senescence candidate genes from the literature.

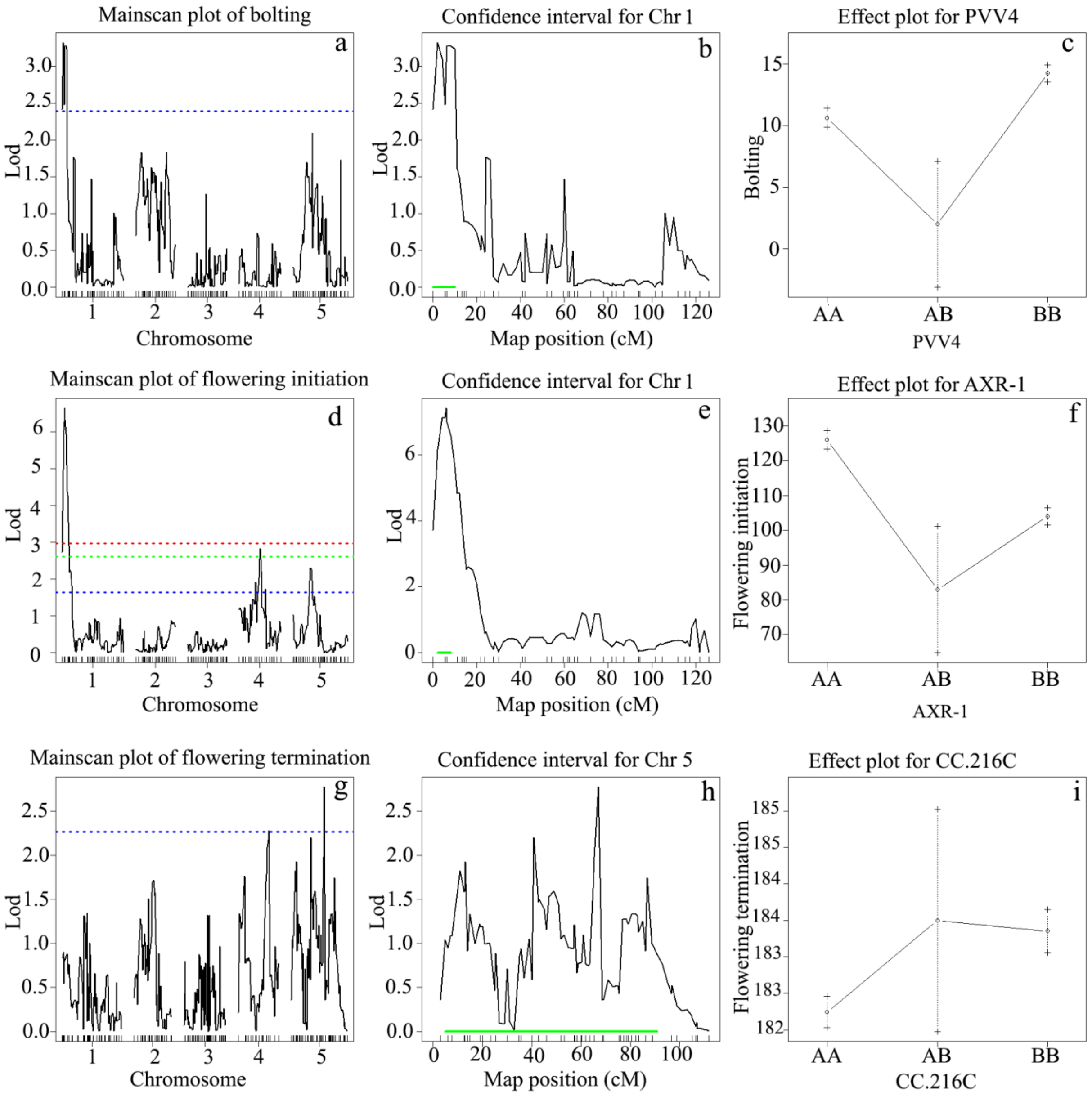

Supplementary Fig. S5|QTL analysis of reproductive traits: Significant LOD scores were determined by permutation testing and are indicated by horizontal lines. Genome-wide scan (main scan), confidence interval for the main chromosome involved (green lines) and allele effect plot of the main effect QTL are shown for Bolting (a-c), flowering initiation (d-f) and flowering termination timing (g-i). In the main scan, red and green dashed lines represent the threshold of P = 0.05, P=0.1 respectively and the blue dashed line represents the threshold for suggestive QTL (P = 0.63). In allele effect plot, number of days from germination to each trait is indicated as mean among ten replicates at each QTL, using the closest marker for each genotype.

Supplementary note:

Candidate genes overlapping all significant and suggestive QTLs

Regarding bolting and flowering initiation traits, *c1.loc2* and *c1.loc6*, overlapped with LHY (LATE ELONGATED HYPOCOTYL), *AXR1* (AUXIN RESISTANT 1), respectively. *c5.loc36* overlapped with the flowering gene *FPF1* (FLOWERING PROMOTING FACTOR 1), and although *c4.loc44* was assigned for flowering initiation, it overlapped with the senescence gene *NYC1* (*NON-YELLOW COLORING 1*). Considering genes involved in senescence, *c4.loc58* co-localized with the *WRKY53* genes and *c5.loc64* with *ANAC0 92* (ARABIDOPSIS NAC DOMAIN CONTAINING PROTEIN 92). Among detected, but non-significant and/or suggestive loci were *c1.loc10*, which was detected for bolting and overlapped with CRY2 (CRYPTOCHROME 2) and *c1.loc40*, detected for both bolting and flowering initiation, and overlapping with PIF3 (PHYTOCHROME INTERACTING FACTOR 3) *and PHYA* (PHYTOCHROME A). *c5.loc56*, assigned for flowering initiation, overlapped with the flowering gene, *PHYC* (PHYTOCHROME C). *c4.loc84*, assigned for both flowering initiation and termination, co-localized with the RCCR gene (RED CHLOROPHYLL CATABOLITE REDUCTASE). *c5.loc90* was assigned for flowering termination and overlapped with the flowering gene *VIN3* (VERNALIZATION INSENSITIVE 3), but was close to two important senescence genes: *SAG113* (*SENESCENCEASSOCIATED GENE 113*) and ANAC100 (NAC DOMAIN CONTAINING PROTEIN 100). Two QTLs, *c4.loc2* and *c5.loc2*, were detected for both flowering initiation and termination. At the moment, no obvious candidate senescence gene can be assigned to this locus. But these overlap with flowering genes FRI (FRIGIDA) and PNY (PENNYWISE), respectively.

A suggestive QTL: *c1.loc2* was identified for bolting time, and it overlaps with the LHY gene. LHY participates in the photoperiod and circadian clock flowering pathways. *lhy* mutants have shown early flowering in short days (Koornneef et al, 1991; Alabadi et al, 2001; Mizoguchi et al, 2002). Another locus detected for bolting (even though non-significant) was c1.loc10, which overlapped with the flowering gene CRY2. CRY2 encodes a protein with a blue light photoreceptor in the light perception flowering pathway. Late flowering has been shown in cry2 mutants (Guo et al, 1998; Ni et al, 1998; El-Assal et al, 2001).

Even though *c1.loc8* and *c5.loc56* (associated with flowering initiation) didn't show significant effects, they were co-localized with flowering genes PIF3& PHYA and PHYC, respectively. PIF3 encodes a bHLH transcription factor in the photoperiod and circadian clock flowering pathways. Antisense suppression of PIF3 causes early flowering (Oda et al, 2004; Johnson et al, 1994). PHYA codes for a red or far-red light photoreceptor in the light perception flowering pathway. Late flowering has been shown in *phya* mutants in long days (LD) and overexpression of this gene promotes early flowering in short days (SD) and LD (Bagnall et al, 1995; Franklin et al, 2003). The PHYC gene also codes for the circadian clock flowering pathway and mutants of *phyc* show early flowering in short days (Monte et al, 2003; Koyama et al, 2013).

Three QTLs (*c4.loc2*, *c4.loc84* and *c5.loc2*) were assigned to both flowering initiation and flowering termination, and they overlap with the flowering gene, FRI, the senescence gene, RCCR, and the flowering gene, PNY, respectively. In addition, *c5.loc90*, which was associated with flowering termination time, was co-localized with the flowering gene, VIN3. The closest senescence gene to this locus is SAG113, encoding a member of the PP2C (PROTEIN PHOSPHATASE 2C) family, controlling dehydration in senescing leaves (Wingler et al, 2010).

Overlapping flowering and senescence genes with loci affecting either of those traits suggest that reproductive traits can depend on each other. Furthermore, no significant QTL for bolting was associated with these two loci. This relationship is consistent with regulation of flowering time by leaf senescence, previously reported in the Bay×Shadhara population (Schaffer et al, 1998). RCCR has been shown to be essential for chlorophyll degradation. In rice, Knockdown of OsRCCR1 resulted in leaf death and lesion mimic spots (Tang et al, 2011). The described roles of WRKY53, RCCR, and ANAC092 are thus consistent with the effects of QTLs detected for flowering termination. Further experiments are required to determine whether these candidate genes control the observed phenotypic variants and to identify molecular mechanisms involved (modification of the coding sequence in expression level or epigenetic regulation).
